# Identification of potential serum peptide biomarkers of biliary tract cancer using MALDI MS profiling

**DOI:** 10.1186/1472-6890-14-7

**Published:** 2014-02-04

**Authors:** Neomal S Sandanayake, Stephane Camuzeaux, John Sinclair, Oleg Blyuss, Fausto Andreola, Michael H Chapman, George J Webster, Ross C Smith, John F Timms, Stephen P Pereira

**Affiliations:** 1 UCL Institute for Liver and Digestive Health, Royal Free Hospital Campus, University College London, London, UK; 2 Cancer Proteomics Laboratory, EGA Institute for Women’s Health, University College London, London, UK; 3 Department of Gastroenterology, University College Hospitals NHS Foundation Trust, London, UK; 4 Kolling Institute, University of Sydney, St Leonards, Australia

**Keywords:** Cholangiocarcinoma, Biliary tract cancer, PSC, Serum peptide biomarker, MALDI-TOF MS

## Abstract

**Background:**

The aim of this discovery study was the identification of peptide serum biomarkers for detecting biliary tract cancer (BTC) using samples from healthy volunteers and benign cases of biliary disease as control groups. This work was based on the hypothesis that cancer-specific exopeptidases exist and that their activities in serum can generate cancer-predictive peptide fragments from circulating proteins during coagulation.

**Methods:**

This case control study used a semi-automated platform incorporating polypeptide extraction linked to matrix-assisted laser desorption/ionisation time-of-flight mass spectrometry (MALDI-TOF MS) to profile 92 patient serum samples. Predictive models were generated to test a validation serum set from BTC cases and healthy volunteers.

**Results:**

Several peptide peaks were found that could significantly differentiate BTC patients from healthy controls and benign biliary disease. A predictive model resulted in a sensitivity of 100% and a specificity of 93.8% in detecting BTC in the validation set, whilst another model gave a sensitivity of 79.5% and a specificity of 83.9% in discriminating BTC from benign biliary disease samples in the training set. Discriminatory peaks were identified by tandem MS as fragments of abundant clotting proteins.

**Conclusions:**

Serum MALDI MS peptide signatures can accurately discriminate patients with BTC from healthy volunteers.

## Background

Cholangiocarcinoma (CCA) is a tumour which arises from biliary epithelium within the liver (intrahepatic CCA) or from the extrahepatic bile ducts (extrahepatic CCA). Gallbladder carcinomas together with CCA are often grouped as biliary tract cancer (BTC) [1]. CCA is the second most common liver tumour, accounting for about 10% of primary liver malignancies although the incidence is rising [2,3]. Primary sclerosing cholangitis (PSC) can cause benign biliary stricturing and is the most common risk factor for CCA in Western populations, with rates of 8-30% reported in clinical follow-up or liver explant specimens after transplant. The prognosis of CCA is poor with an overall median reported survival of less than 1 year after diagnosis [4]. Surgery is the only curative treatment option at this time, with resectability rates ranging from 36% to 77% [5,6]. Serum carbohydrate antigen (CA19-9) has been extensively studied and is the most common tumour marker used in assisting in the diagnosis and monitoring of CCA [7]. However, CA19-9 can also be raised in benign biliary obstruction and cholangitis as well as other gastrointestinal and gynaecological neoplasms [8], and is virtually undetectable in the 7% of the population who are negative for the Lewis antigen [9]. More accurate circulating biomarkers in this aggressive and fatal cancer are clearly needed.

In recent years, proteomic profiling strategies using automated magnetic reverse-phase beads for analyte capture and matrix-assisted laser desorption/ionisation time-of-flight (MALDI-TOF) MS have reported apparent disease-specific proteins and peptides for bladder, pancreatic and gastric cancers [10-13]. Further work to identify the serum peptides suggest that they are mostly fragments derived from endogenous proteins of high abundance that may originate from protein breakdown products of the clotting cascade formed *ex vivo* by cancer-specific exoproteases [13].

The aim of this discovery study was to profile peptides in serum collected from patients with BTC, patients with benign biliary disease and healthy volunteers using magnetic C18 bead-based, solid-phase polypeptide extraction followed by MALDI-TOF MS profiling. Predictive models based on differential peptide peaks generated from a training set were tested on an independent validation set of samples and a subset of the discriminant peptides were identified by liquid chromatography-tandem mass spectrometry (LC-MS/MS).

## Methods

### Patients and serum samples

This study was approved by the Joint UCL/UCLH Committees on the Ethics of Human Research (Committee A) (Reference No. 06/Q0152/106). Sample collection followed institutional Ethical Committee guidelines in accordance with the principles outlined in the Declaration of Helsinki and patient informed consent was obtained. Serum samples were prospectively collected from 92 patients diagnosed with BTC and benign biliary strictures or healthy volunteers attending University College London Hospital between 2006 and 2008. The BTC and benign patients were a heterogeneous mix of patients none of whom were receiving active chemotherapy or antibiotics for cholangitis at the time of sampling. Healthy volunteers had no active illnesses and were not on medication and were comprised of relatives who accompanied the BTC and benign patients to the clinic. A further set of 30 BTC and healthy volunteer samples were also prospectively collected in 2009/2010 as an independent validation set. Blood samples were collected in 8.5 mL BD Vacutainer® SST™ Advance Tubes (Becton Dickinson Diagnostics, New Jersey), gently inverted 5 times and clotted at room temperature for 60 min. Samples were then centrifuged at 2,200 rpm at 4°C for 12 min and serum supernatant aliquoted into cryovials and frozen at -80°C until further use. This standard operating procedure (SOP) was used for all samples with those from healthy volunteers processed at the same time as their relatives’ case samples. Patients with BTC were histologically and/or cytologically proven to have adenocarcinoma and were staged according to the tumour-node-metastasis (TNM) system for either CCA or gallbladder carcinoma [14]. Patients with benign biliary disease had a range of diagnoses including PSC, autoimmune pancreatitis/IgG4-associated cholangitis (AIP/IAC), papillary stenosis/fibrosis, chronic pancreatitis and sphincter of Oddi dysfunction. Serum liver biochemistry, C-reactive protein (CRP) and serum CA19-9 assays (electro-chemiluminescence immunoassay; Roche Modular) were performed for all patients with malignant and benign biliary disease. A CA19-9 value of >37 IU/mL was considered as abnormal, as reported in several other studies [7]. Tables 1 and 2 outline the clinical, demographic and biochemical characteristics of the patients used in the discovery phase of this study.

**Table 1:** Demographics and biochemical profile of the patient cohort used for biomarker discovery

	**Clinical group**				
**Variable**	**BTC**	**PSC**	**IAC**	**Benign other**	**Healthy**
Number	39	10	7	14	22
Female: male	15:24	3:7	0:7	9:5	7:15
Age (yrs)	69 (27–93)	48 (22–76)	64 (43–71)	53 (35–47)	60 (39–78)
Bilirubin (μmol/L)	40 (8–616)	17 (7–457)	12 (5–40)	8 (4–38)	ND
CA19-9 (U/mL)	295 (1–145528)	17 (1–4119)	15 (1–52)	ND	ND
CA19-9 >37 U/mL	30/39	3/10	1/4	ND	ND
CRP mg/L (N < 5)	44.4 (1–171)	9.9 (1–194.2)	8.6 (5–35.7)	ND	ND
ALP (U/L)	577 (138–1925)	195 (98–514)	229 (69–642)	ND	ND
IgG4 g/L (N < 1.3 g/L)	ND	ND	1.5 (0.49-2.57)	ND	ND
WCC (x10^9^/L)	8.2 (3.3-14.8)	6.0 (4.3-15.0)*	7.6 (2.3-9.8)	ND	ND
Neutrophils (x10^9^/L)	5.9 (2.3-12.0)	3.3 (2.8-13.4)*	4.1 (0.7-5.4)	ND	ND

BTC, biliary tract cancer; PSC, primary sclerosing cholangitis; IAC, IgG4-associated cholangitis. Benign other group comprises sphincter of Oddi dysfunction, stenosis of the Ampulla of Vater and chronic pancreatitis. CA19-9, carbohydrate antigen 19–9; CRP, C-reactive protein; ALP, alkaline phosphatase; IgG4, immunoglobulin G4; WCC, white cell count. Unless otherwise indicated, values indicate median (with range); ND, not determined; *WCC and neutrophil counts were available for only 6 of the 11 PSC patients.

**Table 2:** Demographics, clinical and pathological characteristics of biliary tract cancer patients

Total number of BTC patients	39
Males	24
Females	15
Cholangiocarcinoma	37
Gallbladder carcinoma	2
Positive cytology	17
Positive histology:	22
Poorly-differentiated	11
Moderately-differentiated	10
Well-differentiated	1
Intrahepatic cholangiocarcinoma	2
Extrahepatic cholangiocarcinoma:	35
Hilar	31
Distal bile duct	4
Stage:	
T1 or T2	18
T3 or T4	21
Deaths	33/39 (85%)
Median survival and range (months)	8.4 (1.4-43.9)

### Polypeptide extraction and sample preparation

One aliquot of serum per subject was thawed and 3 × 50 μL randomly distributed into three replica 96-well plates. Plates were again stored at -80°C prior to running on separate days. Serum polypeptides were extracted, mixed with matrix and spotted onto MALDI target plates using a semi-automated C18-coated magnetic bead-based extraction and spotting protocol [15] (described in detail in Additional file 1). The triplicate extracts from each sample were spotted in quadruplicate onto MALDI target plates to generate 12 spotting replicates per subject sample. For each target plate, four replicate samples of a quality control human serum (Sigma-Aldrich, Dorset, UK; #S7023) were extracted and run alongside case control samples to monitor inter-assay variation.

### MALDI-TOF MS data acquisition and processing of spectral data

Spectral profiles were acquired automatically using an Ultraflex MALDI-TOF/TOF mass spectrometer (Bruker Daltonics, Bremen, Germany) and data quality filtering applied essentially as described [15] (see Additional file 1 for details). ClinProTools (CPT) version 2.2 (Bruker Daltonics) was used to process spectral data. Average peak areas and standard deviations for the normalised data were calculated for each clinical group (BTC, benign and healthy) prior to statistical analysis.

### Statistical analysis and validation

A peak list containing all spectra classified into groups comparing BTC with healthy controls, and BTC with benign, was generated in CPTv2.2. The *m/z* values representing the peaks were filtered by first applying an Anderson-Darling test to assess the distribution of peak areas. Significance in the change in average peak area was then assessed using either a Student *t*-test (Anderson-Darling *P* > 0.05 = normal distribution) or Wilcoxon test (Anderson-Darling *P* < 0.05 = non-normal distribution). Calculations accounted for the consequences of multiple testing by using the Benjamini-Hochberg *P*-value adjustment. Receiver Operating Characteristic (ROC) curves were generated to compare sensitivity and specificity of discriminating peaks. Classification models were generated from a training set consisting of 75% of the BTC and healthy samples using the model generation function of CPTv2.2. QuickClassifier, Supervised Neural Network, Support Vector Machine (SVM) and Genetic Algorithms were tested and 20% leave-out cross validation (LOCV) was carried out to select the best models for testing on the remaining 25% of samples. Classification performances were then compared. Simple models with defined thresholds were also generated using the discriminatory peaks. For this, a subroutine was written in Matlab to check all combinations of peaks using normalised peak areas or log-transformed peak areas with various degrees of power between -2 and 2 for each peak. Model classification performances were then compared.

A second set of serum samples was prospectively collected from patients with BTC (n = 14) and healthy volunteers (n = 16) (Additional file 2: Table S1). Sample collection, handling and profiling methodology were identical. The aim of this phase of the study was to validate the models generated in the discovery phase by assessing sensitivity, specificity, positive and negative predictive values.

### Identification of MALDI-TOF peptides/peaks

Discriminatory peaks were selected for identification by LC-MS/MS. Individual BTC and healthy control samples were selected for pooling based on the highest areas for these peaks. Pools (400 μL total volume) were extracted manually with 40 μL of RPC18 Dynabeads (50 mg/mL) per pool and using 56 μL of elution buffer (50% acetonitrile/0.1% TFA). Eluates were split into two and dried in a SpeedVac. One sample was resuspended in phosphate buffered saline (PBS) and run on a 10-20% 1.0 mm Tricine gel and stained with InstantBlue (Novexin). Gel bands (n = 12) were cut below the 20 kDa marker, destained by shaking at room temperature for 30 min in 200 μL of 50% methanol/10% acetic acid and then washed in 100 μL of 100% acetonitrile with shaking for 15 min. Polypeptides were then extracted in 200 μL of 50% formic acid, 25% acetonitrile, 15% isopropanol by vigorous shaking for 2 hrs at room temperature. The extract was recovered, dried down and resuspended in 0.1% formic acid. One fifth of this sample was analysed by MALDI-TOF MS as described above, to verify the presence of peaks of interest. The remainder of the sample was subjected to ZipTip clean-up and analysed by LC-MS/MS. The other extracted sample was resuspended in 0.1% formic acid and analysed directly by LC-MS/MS. Details of the LC-MS/MS analysis are provided in Additional file 1.

## Results

### Detection of MALDI-TOF peaks discriminating BTC and healthy controls

**Figure 1:**
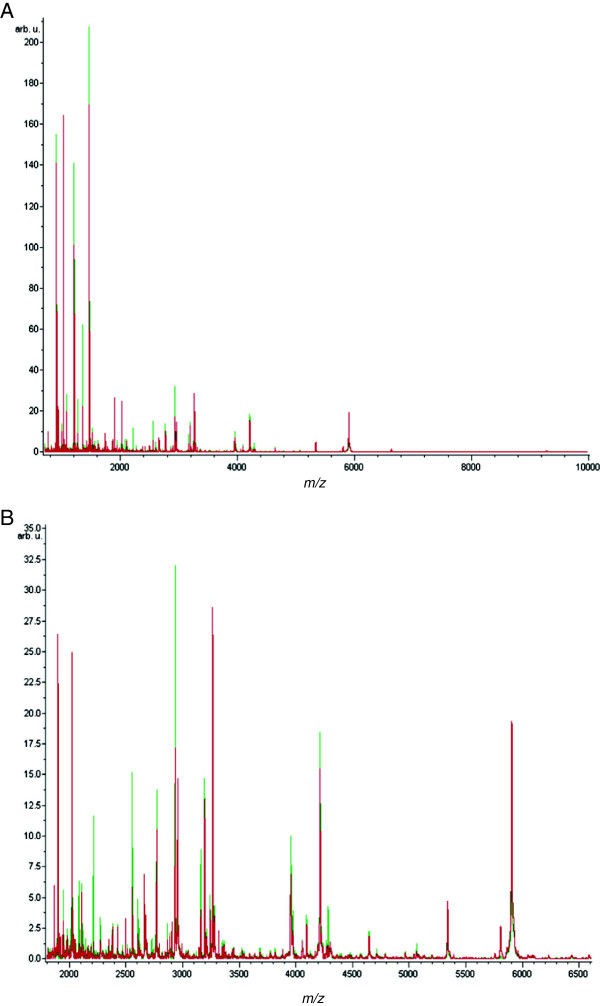
**MALDI MS profiling of serum peptides in biliary disease. A**. Average MALDI-TOF spectra for BTC (red) and healthy (green) serum samples over the full scan range of *m/z* 700–10,000. **B**. Zoomed spectra over the *m/z* 1,700 to 6,900 range.

**Figure 2:**
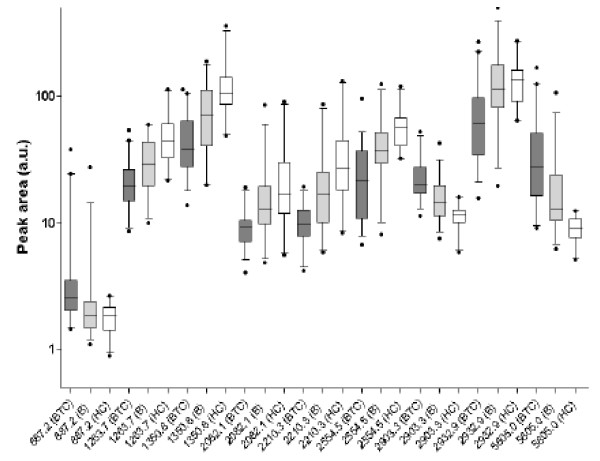
**Peaks discriminatory for BTC versus benign biliary disease and healthy controls.** Box-and-whisker plots of peak area (5–95 percentile) are shown for differential peptide peaks for biliary tract cancer BTC; dark grey), benign disease (B; light grey) and healthy control (HC; white) samples.

**Figure 3:**
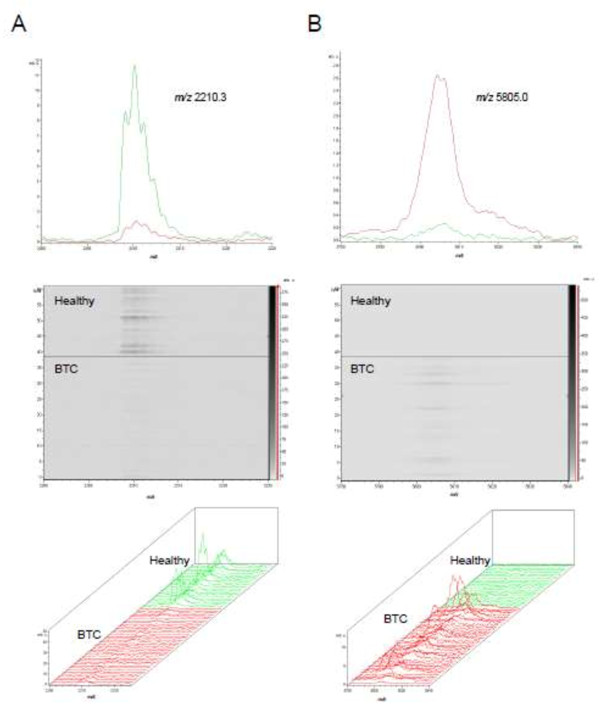
**Average spectra, gel and 3D spectral views for two significant peaks discriminating BTC (red) and healthy control (green) samples. A**. The peak at *m/z* 2212.3 was generally of lower intensity in BTC samples compared to healthy samples. **B**. The peak at *m/z* 5805.0 was generally of higher intensity in BTC samples compared to healthy samples.

Ninety two serum samples comprising patients with BTC (n = 39), benign biliary disease (n = 31), and healthy controls (n = 22) were subjected to C18 reversed-phase extraction in triplicate and analysed by MALDI-TOF MS profiling. Assay performance was assessed within and across runs using a quality control serum sample processed and spotted multiple times with test samples. The profiling method gave an inter-assay CV of 12.8% +/- 6.7 using all detected peaks. For the discovery samples, 303 aligned peaks were detected in the *m/z* range 700–10,000 (Figure 1). Using a conservative *P* value cut-off of <0.001 and an average-fold change of ≥2.0, eight peaks discriminated the BTC and healthy groups (Table 3 and Additional file 2: Table S2). Peaks *m/z* 887.2, 2903.3 and 5805.0 were up-regulated in BTC samples, whereas peaks *m/z* 1263.7, 1350.8, 2082.1, 2210.3 and 2554.5 were down-regulated. Box-and-whisker plots for these and an additional peak of interest (*m/z* 2932.9; *P* = 0.002) are illustrated in Figure 2. As examples, peaks *m/z* 5805.0 and *m/z* 2210.3 are shown in spectral and gel views in Figure 3. Two-dimensional scatter plots of discriminatory peaks showed the separation of BTC and healthy samples (Additional file 2: Figure S1). In the comparison of the BTC and benign cases, there were fewer discriminatory peaks with seven showing a >1.5-fold change in area with *P* < 0.05 (Additional file 2: Table S3). Six of these peaks were common to both comparisons (*m/z* 887.2, 2082.1, 2210.3, 2554.5, 2903.3 and 5805.0) and displayed the same directionality of change when comparing BTC with healthy and benign (Figure 2). When BTC samples were compared to the benign subset from ten PSC patients, there were no significantly differentiating peaks detected.

**Table 3:** Significant peptide peaks differentiating BTC from healthy volunteers

**Mass (*m/z*)**	**Peak area**	**Peak area**	**Fold- change**	**Wilcoxon test**	**AUROC**^**†**^
	**BTC +/- SD**	**H +/- SD**	**(BTC vs H)**	***P *** **value**	
887.2	5.2+/-7.1	1.8+/-0.5	2.82	0.000691	0.79
1263.7	21.8+/-9.9	47.4+/-22.7	0.46	<0.000001	0.91
1350.8	48.3+/-25.7	117.4+/-62.3	0.41	<0.000001	0.92
2082.1	10.0+/-3.4	24.4+/-20.5	0.41	0.0000364	0.86
2210.3	10.9+/-3.8	38.2+/-31.6	0.29	<0.000001	0.92
2554.5	24.8+/-17.8	57.3+/-20.6	0.43	<0.000001	0.91
2903.3	24.1+/-10.0	11.5+/-2.5	2.09	<0.000001	0.97
5805.0	33.0+/-30.9	6.7+/-1.6	4.93	<0.000001	0.96

*Wilcoxon test *P* values were corrected for multiple testing using the Benjamini-Hochberg adjustment. ^**†**^Area under the Receiver Operating Characteristic curve.

Based on the quantitative data of the discovery set for discriminating BTC from healthy volunteers, the area under the ROC curves (AUROC) for peaks *m/z* 2903.3 5805.0, 2210.3 and 1263.7 were 0.97, 0.96, 0.92 and 0.91, respectively (see Table 3 and Additional file 2: Figure S2, for example ROC curves). For BTC versus benign cases, AUROC values for the seven discriminatory peaks were in the range 0.73 to 0.78 (Additional file 2: Table S3).

### Class prediction model generation and external validation

Seventy five per cent of the BTC and healthy control samples were randomly assigned to a training set and the remaining 25% assigned to a test set. Five permutations of these training and test sets were created. Class prediction analysis was then performed using either the QuickClassifier, Supervised Neural Network, Genetic Algorithms or Support Vector Machine (SVM) modelling in the ClinProTools software with cross-validation using 20%-LOCV (10 iterations). Different models were tested using various numbers of k-Nearest Neighbours (k-NN) and peaks. Four hundred models were generated in total for the five permutated sets. The best performing models from this internal validation are shown in Additional file 2: Table S4. The best was a 9 peak SVM (3 k-NN) model which gave 94.4% accuracy on cross-validation and 100% sensitivity, specificity, accuracy, positive predictive value and negative predictive value for test set prediction. The peaks automatically chosen by this model were *m/z* 1020.2, 1263.7, 1350.8, 1363.4, 2210.3, 2554.5, 2624.5, 2903.3, and 5064.8, and thus included five of the most discriminatory peaks found in the discovery analysis (Table 3). This model was next tested on profiling data from a second independent set of BTC and healthy volunteer serum samples. The sensitivity, specificity, positive and negative predictive value (PPV, NPV) for the model were 92.9%, 100%, 100%, and 94.1%, respectively, and thus discriminated BTC from healthy samples with high accuracy. This model however performed less well when tested for the discrimination of BTC and benign cases.

Since there was no control over the peaks entering the SVM and no thresholds defined, we sought to generate and test simpler models using only the most discriminatory peaks and to define usable thresholds. Peak *m/z* 2903.3 was excluded from this analysis as it was suspected to be a doubly charged form of *m/z* 5805.0 and therefore not an independent molecular species (see below). The best model at discriminating the discovery BTC cases from healthy controls was (5805)/(1350*2210*2554)*1000 > 0.3 (using normalised areas for each peak) with a sensitivity of 97.4% and specificity of 100%. When tested on the independent validation set this model gave a sensitivity, specificity, PPV and NPV of 100%, 93.8%, 92.9%, and 100% respectively, for discriminating BTC versus healthy with an AUROC of 0.995 (95% CI 0.98-1.01), SE = 0.008 and *P* < 0.0001.

For predicting BTC versus benign cases, the best model (((10*log878)*log1350*log5805^1.25)/(log2210^(1.5)*log2554^(0.75)) > 5) gave a sensitivity, specificity, PPV and NPV of 79.5%, 83.9%, 86.1% and 76.5%, respectively, on the training set data, although could not be tested independently due to a lack of available samples from additional benign cases. The most accurate model in discriminating BTC from both healthy and benign controls was (5805)/(2082*2554*2923)*1000 > 0.4, and provided a 92.3% sensitivity and 100% specificity for BTC versus healthy (AUROC = 0.981 (95% CI 0.95-1.01), SE = 0.014, *P* < 0.0001) and 92.3% sensitivity and 74.2% specificity for BTC versus benign (AUROC = 0.815 (95% CI 0.706-0.923), SE = 0.055, *P* < 0.0001)) using the discovery set data.

### Peak identification

**Figure 4:**
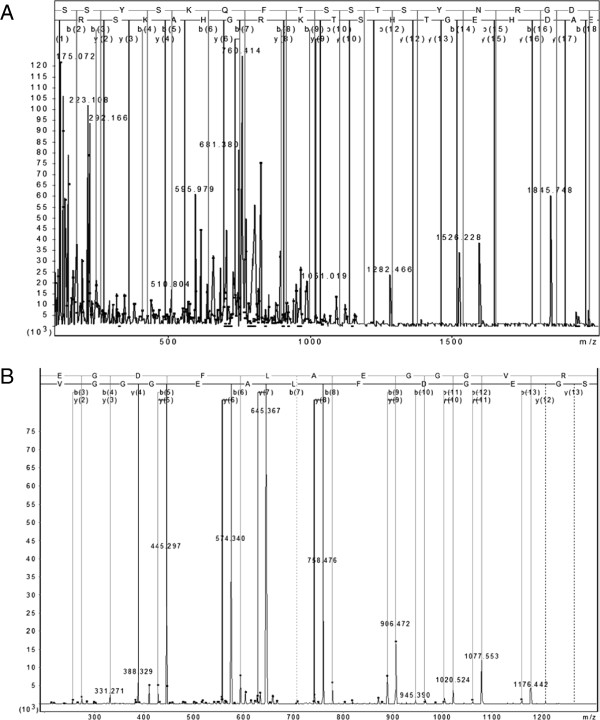
**MS/MS fragmentation and sequence data for identified peptides. A**. The *m/z* 5805.0 peak (SSSYSKQFTSSTSYNRGDSTFESKSYKMADEAGSEADHEG-THSTKRGHAKSRP) was identified as a fragment of isoform 1 of fibrinogen alpha chain (IPI00021885) with an ion score of 122 from *m/z* [726.214]^8+^. The spectrum shown is the sum of 28 scans in range 130 (rt = 22.952) to 198 (rt = 31.9511). **B**. The *m/z* 1350.8 peak (SGEGDFLAEGGGVR) was also identified as a fragment of fibrinogen alpha chain with an ion score of 95 from *m/z* [675.82]^2+^.

Discriminatory peaks used in the models were targeted for identification by LC-MS/MS following C18 extraction and separation on high-percentage 1D gels (Additional file 2: Figure S3). Four of 13 peaks of interest were identified, three of which have been reported in other studies, adding confidence to the identifications (Figure 4 and Additional file 2: Table S5). The identities of five more peaks were inferred from previous studies [13,16] including our own [17], whilst the remaining peaks eluded identification. Most of the identified and predicted peptides are derived from fibrinogen alpha, with a peptide also identified from the abundant serum protein kininogen. These peptides are hypothesized to be generated through the actions of blood-borne clotting endopeptidases and as yet uncharacterised exopeptidases [13].

## Discussion

In this study we identify serum peptide peaks as potential markers of BTC utilising a high-throughput C18 bead-based extraction method linked to MALDI-TOF MS profiling and LC-MS/MS for subsequent peak identification. A predictive model using an SVM consisting of nine peaks was able to distinguish BTC from healthy controls in a set of independent validation samples with a high degree of accuracy. Several of these same peptides could also distinguish BTC from benign cases of biliary disease albeit with lower accuracy, though none were discriminatory when using a smaller subset of 10 samples from patients diagnosed with PSC.

Serum profiling using proteomics has developed significantly from its early years when it was claimed that surface-enhanced laser desorption ionisation time-of-flight mass spectrometry (SELDI-TOF MS) profiling could reveal discriminatory peaks of high diagnostic accuracy for various cancers [18-22]. Following the initial excitement, scepticism about the methodology mounted with reports of experimental bias within datasets and evidence that variations in sample handling gives rise to “differential” peptides previously reported as cancer markers [23-27]. Our study was designed prior to sample collection, and hence all samples underwent the same process of prospective collection and were handled using a robust SOP. These aspects are crucial to avoid variability in profiling particularly as the peptides of interest are thought to be generated from products of the clotting cascade *ex vivo*.

Our main aim was to identify low molecular weight polypeptides in serum for detecting BTC using healthy volunteers and benign cases of biliary disease as control groups. This work was based on the hypothesis that cancer-specific exoproteases in serum act to digest products of the clotting cascade *ex vivo*, generating peptide fragments which may discriminate cancer from healthy samples [13,28,29]. As such, serum, rather than plasma was chosen for this study. Analysis of spectral profiles from 92 serum samples gave eight significant peaks (*P* < 0.001) with a >2.0-fold change in peak area between BTC patients and healthy subjects. Two of these peaks (*m/z* 5805.0 and *m/z* 2903.3) had higher peak areas in BTC versus healthy, whilst the other six peaks were higher in the healthy samples. The best model generated from the discovery data using several of these peaks gave 100% sensitivity, 93.8% specificity, 92.9% PPV and 100% NPV when tested on the independent validation dataset, showing the potential of the test for accurate diagnosis of BTC.

Biliary strictures can arise from several non-malignant conditions which can mimic BTC, such as AIP/IAC, PSC and chronic pancreatitis. There were seven significant (and overlapping) peaks discriminating samples from patients with BTC versus benign biliary disease and a model was generated from the discovery data with respectable diagnostic accuracy (79.5% sensitivity, 83.9% specificity), These peaks, particularly *m/z* 5805.0, may therefore be of utility in differentiating BTC from benign disease. Despite this, the profiling failed to accurately differentiate patients with BTC versus the small subset of patients with PSC – a risk factor for BTC [30]. This may be due to the low number of samples used or the fact that the identified peaks may be markers of inflammation of the biliary epithelium. Indeed, the median level of C-reactive-protein (CRP) was significantly higher in the BTC patients than in the PSC cohort (Table 1). Whilst our preliminary findings would argue against this, further independent validation using greater numbers of samples from patients with PSC and other benign conditions will be necessary to determine the robustness of this model for the differential diagnosis of BTC. It is noteworthy that other cancer biomarker discovery studies using similar methodologies have not included benign inflammatory groups [13,31] and it is now becoming evident that this is absolutely critical. One recent study in pancreatobiliary disease has addressed this issue, with the study identifying a potential marker of malignancy in bile (NGAL) that was independent of markers of biliary obstruction and inflammation [32]. As in the present study, further validation of this marker is required.

An ion of mass [726.214]^8+^ was identified as the 576–628 fragment of isoform 1 of fibrinogen alpha chain by LC-MS/MS with a calculated average mass of 5805.09 Da and we matched this to the MALDI-TOF peak at *m/z* 5805.0. This peak may be generated by the exopeptidase-mediated loss of valine from the abundant peak at *m/z* 5903.9, which has been identified as a large fragment of fibrinogen alpha [17]. It may thus represent a surrogate marker of a tumour-derived exoprotease that is present at higher levels in the blood of BTC patients. The lower intensity peak at *m/z* 2903.3 behaved similarly to *m/z* 5805.0, but could not be identified. Its mass, isotopic pattern and expression behaviour suggest it to be a doubly-charged form of *m/z* 5805.0. As such, both peaks cannot be used as independent discriminating features, although we note that only the peak at *m/z* 2903.3 was selected by the SVM model used for validation. The other discriminatory peaks were also identified as fragments of fibrinogen alpha/fibrinopeptide A and high molecular weight kininogen, which have been reported as surrogate markers of different cancer types [13].

Exoproteases form a heterogeneous group of enzymes that play a role in the regulation of biologically active peptides. Examples such as leucine aminopeptidase, aminopeptidase A, aminopeptidase N, carboxypeptidase N and the kininase I family of carboxypeptidases are involved in the production of angiotensin, bradykinin and vasopressin [33], whilst carboxypeptidase B2 is involved in the down-regulation of fibrinolysis [34]. Through such activities, they may also contribute to tumour progression and invasiveness. In particular, several studies have reported the elevated expression of aminopeptidase N/CD13 in various cancers [35-38] and it is believed to play a role in angiogenesis [39]. We speculate that the peptide fragments detected in our study are likely to be generated by such exopeptidase activities and thus serve as surrogate markers of the exoproteases themselves. The identity of these proteases was not the focus of this study, but their future interrogation in BTC may shed further light on its pathophysiology and lead to the identification of tumour-specific biomarkers and possible targets for therapy.

Various fibrinogen alpha chain and fibrinopeptide A fragments have been detected in hepatocellular, ovarian, urothelial and gastric cancers [13,40-43], although the *m/z* 5805.0 fragment has not been identified previously. These cleavage products may therefore be indicative of an underlying malignancy rather than be specific for BTC. However, we do note that our group could not derive a peptide signature to accurately differentiate ovarian cancer from benign ovarian disease or healthy controls using serum peptides detected on the same profiling platform [15]. This suggests that the signature identified herein, displays some specificity for BTC.

There are several limitations to the present study. The SOP adopted for this study used a clotting time of 60 minutes and therefore may be difficult to translate into the clinical laboratory where time and staff limitations are restrictive. In addition, the extraction and MALDI-TOF MS profiling method used here requires considerable expertise for operation and determines only relative peptide quantities. Given the nature of the peptides, the generation of fragment-specific antibodies for use in immune-based assays may be problematic and so MS-based targeted assays using synthetic, stable isotope-labelled peptide standards for accurate quantitation would be a more attractive way to validate our findings. Finally, the validation would require larger sample numbers, particularly those from cases of benign biliary disease.

## Conclusions

In conclusion we have defined a serum peptide signature and model than can accurately discriminate patients with BTC from healthy volunteers and which was validated on an independent sample set. A model using an overlapping set of peptides was also derived that could differentiate malignant from benign disease, albeit with a lower, but respectable, accuracy. Further independent validation of this model is now required using greater numbers of samples from patients with PSC and other benign conditions to test its robustness for the differential diagnosis of BTC.

## Abbreviations

AIP: Autoimmune pancreatitis; AUC: Area under the ROC curve; BTC: Biliary tract cancer; CA19-9: Carbohydrate antigen 19–9; CCA: Cholangiocarcinoma; CRP: C-reactive protein; LC-MS/MS: Liquid chromatography-tandem mass spectrometry; IAC: Immunoglobulin G4-associated cholangitis; MALDI-TOF: Matrix-assisted laser desorption ionisation time-of-flight; MS: Mass spectrometry; PSC: Primary sclerosing cholangitis; ROC: Receiver operating characteristic; SOP: Standard operating procedure; SVM: Support vector machine; TNM: Tumour-node-metastasis.

## Competing interests

The authors declare that they have no competing interests.

## Authors’ contributions

NSS helped to carry out the MALDI-TOF MS profiling and data analysis, collected clinical specimens and drafted the manuscript. SC carried out the MALDI-TOF MS profiling. JS carried out the LC-MS/MS peak identification. OB carried out statistical analysis and model building. FA, MHC and GJW collected clinical specimens. RCS assisted with statistical analysis. JFT designed and coordinated the study and helped draft the manuscript. SPP conceived the study and helped to draft the manuscript. All authors read and approved the final manuscript.

## Pre-publication history

The pre-publication history for this paper can be accessed here:

http://www.biomedcentral.com/1472-6890/14/7/prepub

## Supplementary Material

Additional file 1 Supplementary Methods. Click here for file

Additional file 2 Supplementary Tables and Figures. Click here for file
