# Supplementary material for: Identification of potential serum peptide biomarkers of biliary tract cancer using MALDI MS profiling
Source: BMC Clin Pathol. 2014 Feb 4;14:7. doi: 10.1186/1472-6890-14-7 (PMC3923428; doi:10.1186/1472-6890-14-7)
Supplement: Additional file 1 — Supplementary Methods. [file 1472-6890-14-7-S1.pdf]

Supplementary Information

Serum polypeptide extraction and sample preparation

One 200 µL aliquot of serum per patient was thawed following initial storage, then 50 µL distributed into three replicate 96-well plates. The location of each sample was changed for each replica plate. The plates were again stored at -80ºC until each plate was run on separate days, ensuring a uniform number of two freeze-thaw cycles for all samples. Serum polypeptides were extracted and spotted onto MALDI target plates using a previously reported semi-automated C18-coated magnetic bead-based extraction protocol (Timms *et al*, 2010). The protocol utilised RPC18 Dynabeads (Invitrogen Ltd, Paisley, UK), which are paramagnetic, non-porous particles modified with hydrophobic C18 reversed phase chromatographic material. Magnetic beads were washed and resuspended in 0.1% trifluoroacetic acid (TFA) to a concentration of 50 mg/mL. All steps were semi-automated on a Genesis Freedom 200 liquid-handling work station (Tecan UK Ltd, Reading, UK). The procedure was carried out in 96-well Star PCR raised rim skirted plates (Starlab (UK) Ltd, Milton Keynes, UK). Magnetic beads were resuspended by pipetting up and down 10 times. A volume of 5 µL of bead suspension was transferred to wells containing the 50 µL aliquot of serum. The sample was mixed by pipetting up and down 10 times and then allowed to stand for 1 min. Beads were then pulled to the side of the well using a magnetic side holder and allowed to settle on the side for 30 sec. The supernatant was removed and discarded. A volume of 200 µL of wash solution (0.1% TFA) was added, the beads were pulled left to right 10 times on a magnetic side holder and then allowed to settle on one side. The washing solution was subsequently removed and the washing step was repeated. Total removal of the washing solution was achieved using a multi-channel pipette. The beads were spun to the bottom of the wells by centrifugation at 2,000 g for 2 min. A

volume of 7 µL of elution solvent consisting of 50% acetonitrile (Rathburn Chemicals Ltd, Walkerburn, Scotland) in 0.1% TFA was added to the bead pellet and mixed by pipetting up and down 10 times. The mixture was left to stand for 30 sec. The 96-well plate was then transferred to the magnetic side holder and the beads were pulled to the side for 30 sec. An aliquot of 35 µL of pre-prepared α-cyano-4-hydroxycinnamic acid (CHCA) in 36%/56%/8% methanol/acetonitrile/water was added to the eluate and mixed by pipetting up and down 5 times. Volumes of 1 µL of the eluate/matrix mix were then spotted in quadruplicate onto a ground steel MALDI target plate (Bruker Daltonics, Bremen, Germany) and the samples were allowed to dry at room temperature. This generated 12 spotting replicates per sample. For every target plate, four replicate samples of a quality control human serum (Sigma-Aldrich Company Ltd, Dorset, UK; #S7023) were extracted and run alongside case control samples to monitor inter-assay variation.

MALDI-TOF MS data acquisition

Spectral profiles were acquired using an Ultraflex MALDI-TOF/TOF mass spectrometer (Bruker Daltonics, Bremen, Germany) essentially as described previously (Timms *et al*, 2010). The instrument was controlled by Flexcontrol v2.0 software (Bruker Daltonics) and equipped with a 337 nm nitrogen laser, a gridless ion source, delayed-extraction (DE) electronics, a high-resolution timed ion selector and a 2 GHz digitizer. The instrument was calibrated using a mix of commercial peptide and protein calibration standards (Bruker Daltonics; Cat. #206195 & #206355; 30 fmol of each peptide, 500 fmol of each protein) prepared in the same matrix solution as above. Spectra were acquired automatically in a 700-10,000 mass-to-charge (m/z) range in linear mode geometry under 25 kV of ion acceleration (with a DE potential

difference of 1.4 kV), 5.9 kV lens potential and high gating strength to deflect ions below 400 m/z. DE was applied at 50 ns to give appropriate time-lag focusing after each laser shot and the sample rate was 2 GS/s. An irradiation program and data quality filtering were automated using FlexControl's 'AutoXecute' function. Briefly, each spectrum was the sum of 1,000 laser shots per spotted sample, delivered to different locations on the spot in 10 sets of 100 shots (at a laser frequency of 10 Hz) after pre-irradiation with 10 shots at 5% higher laser power to improve spectral quality. Evaluation parameters were set so that only spectra (of 100 shots) containing at least one peak with a resolving power of greater than 300 and a signal-to-noise ratio (S/N) >10 in the m/z range of 700-4000 were accumulated. Data were accepted if the sample had at least 2 of the 4 spotting replicates summing up 1000 shots in at least 2 of the 3 run replicates per sample.

Processing of spectral data

ClinProTools (CPT) version 2.2 (Bruker Daltonics) was used to process spectral data. Smoothing was applied by averaging the intensities within a 5-point width moving window followed by baseline subtraction using an algorithm based on finding the lowest points between dominant local intensity maxima within a particular mass window. Normalisation was performed by dividing the intensity of each data point in a spectrum by the sum of all intensities in that spectrum. Intensity values were multiplied by a constant C (C = 2 x 10^7). Peak definition was achieved by identifying all local maxima in the mass spectra above a normalised intensity threshold of 0.2 and signal-to-noise ratio of 3. Peak alignment was next performed without discriminating cases and controls to find common peaks across samples using a mass window of 1500

ppm. Finally average peak areas and standard deviations were calculated for each clinical group; BTC, benign (PSC + IAC + others) and healthy.

Identification of MALDI-TOF peptides/peaks by LC-MS/MS

For LC-MS/MS, 5 µL of sample was injected on to a 300 µm i.d. x 5 mm C18 PepMap guard column (5 µm bead size, 100 Å pore size, LC Packings, Netherlands) and washed for 3 min with 95% solvent A (water + 0.1% FA) at a flow rate of 25 µL/min using an Ultimate 3000 LC system (Dionex). Reversed phase chromatography was carried out on the same system using a 75 µm i.d. x 150 mm C18 PepMap nano LC column (3 µm, bead size, 100 Å pore size, LC Packings, Netherlands) with a linear gradient of 5-50% solvent B (water/ACN 20%:80% v/v + 0.1% FA). This was coupled to an LTQ Orbitrap XL mass spectrometer (ThermoFisher Scientific) via a PicoView nanospray source (New Objective). Initial precursor ion monitoring was used to identify masses matching the peaks of interest and these masses were targeted by mass and retention time in subsequent runs using high resolution MS/MS in the Orbitrap. Raw spectra were processed with Mascot Distiller V2.2.07 and searched against the human IPI database (v3.53, 73,748 entries). No enzyme was selected for searching with the MS tolerance set to ±10 ppm and the MS/MS tolerance set to 0.1 Da. Oxidation (M), dehydration (N-term C) and deamidation (NQ) were set as variable modifications. Data was also searched in the same way using the ‘de novo sequencing’ function of Distiller.
