# Supplementary material for: Identification of potential serum peptide biomarkers of biliary tract cancer using MALDI MS profiling
Source: BMC Clin Pathol. 2014 Feb 4;14:7. doi: 10.1186/1472-6890-14-7 (PMC3923428; doi:10.1186/1472-6890-14-7)
Supplement: Additional file 2 — Supplementary Tables and Figures. [file 1472-6890-14-7-S2.pdf]

Group	Patients	Gender	Median age (yrs)	Age range (yrs)	Median bilirubin (mg/mL)	Median CA 19.9 (IU/mL)	CA 19.9 >37 IU/mL	BTC stage ≥T3
BTC	14	7F:7M	73	44-90	19	404	11/14	8/14
H	16	8F:8M	34	23-80	ND	ND	N/A	N/A

Table S1 Clinical characteristics of external validation set

Mass (*m/z*)	Average peak area BTC	Average peak area H	SD (BTC)	SD (H)	CV% (BTC)	CV% (H)	*P* value (t test)	*P* value (Wilcoxon)	*P* value (Anderson-Darling)	Fold change (BTC vs H)
708.6	1.79	2.99	1.49	2.43	83.67	81.48	0.111	0.0424	0.000001	0.60
758.7	7.91	1.19	15.14	0.61	191.42	51.48	0.0351	0.00522	0.000001	6.65
763.5	1.72	1.13	0.8	0.61	46.67	54.13	0.0156	0.00739	0.00000925	1.52
887.2	5.16	1.83	7.07	0.45	137.13	24.82	0.0264	0.000691	0.000001	2.82
935.1	27.04	4.44	48.06	2.23	177.7	50.26	0.0264	0.00307	0.000001	6.09
948.9	2.57	1.8	1.36	0.64	53.03	35.44	0.0215	0.026	0.000001	1.43
1020.2	136.02	86.52	58.08	28.77	42.7	33.25	0.00152	0.00621	0.0131	1.57
1077.3	34.51	43.94	11.74	11.4	34.01	25.95	0.0202	0.0268	0.168	0.79
1206.6	161.65	221.72	87.29	73.41	54	33.11	0.0273	0.0239	0.161	0.73
1263.7	21.79	47.4	9.85	22.66	45.21	47.81	0.00152	0.000001	0.00000438	0.46
1350.8	48.33	117.35	25.7	62.26	53.16	53.05	0.00152	0.000001	0.0000992	0.41
1363.4	8.79	11.99	2.02	3.02	22.98	25.21	0.00278	0.000364	0.245	0.73
1531.5	5.19	11.03	1.41	17.02	27.2	154.31	0.237	0.00522	0.000001	0.47
1545.7	8.07	6.33	1.84	1.75	22.74	27.64	0.00759	0.00805	0.756	1.27
1562.5	12.34	15.21	9.63	4.75	78.1	31.27	0.237	0.0028	0.000001	0.81
1617.2	18.66	24.73	6.33	6.88	33.92	27.83	0.0116	0.0052	0.000223	0.75
1919.1	9.76	6.94	5.72	2.56	58.62	36.93	0.0392	0.0295	0.000001	1.41
1935.2	11.44	7.26	5.4	2.4	47.19	33.09	0.00278	0.00387	0.00000368	1.58
2012.0	10.1	17.67	2.9	8.1	28.74	45.85	0.00516	0.00196	0.000001	0.57
2028.8	10.75	18.53	3.07	9.15	28.53	49.4	0.00791	0.000509	0.000001	0.58
2082.1	9.97	24.43	3.39	20.47	33.94	83.78	0.0202	0.0000364	0.000001	0.41
2094.0	6.03	10.54	1.96	6.85	32.54	64.98	0.0273	0.00421	0.000001	0.57
2116.2	8.72	13.54	2.62	5.49	30.08	40.51	0.00759	0.000867	0.0000929	0.64
2210.3	10.94	38.18	3.82	31.58	34.95	82.71	0.00759	0.000001	0.000001	0.29
2272.4	7.98	12.36	4.19	5.76	52.52	46.61	0.0202	0.0171	0.0000311	0.65
2348.3	13.44	11.06	3.91	2.62	29.14	23.73	0.028	0.0432	0.454	1.22
2467.1	6.34	7.65	2.16	1.37	34.11	17.88	0.026	0.0156	0.541	0.83
2495.5	17.75	12.45	8.18	3.52	46.12	28.3	0.00872	0.033	0.0000823	1.43
2554.5	24.83	57.32	17.77	20.58	71.57	35.9	0.0000414	0.000001	0.00997	0.43
2604.1	13.25	24.66	5.81	11.94	43.82	48.39	0.00516	0.000476	0.0000274	0.54
2617.6	7.14	13.04	4.19	10.43	58.67	79.97	0.0577	0.0239	0.000001	0.55
2624.6	4.88	6.12	1.34	1.32	27.52	21.51	0.00967	0.0171	0.82	0.80
2726.1	11.67	15.04	3.35	4.92	28.69	32.71	0.0316	0.0171	0.00137	0.78
2760.0	9.76	13.03	3.58	4.23	36.69	32.48	0.0218	0.0239	0.00112	0.75
2769.8	46.96	62.19	44.88	32.6	95.57	52.42	0.248	0.0445	0.000001	0.76
2781.7	6.87	8.29	3.33	2.4	48.4	28.99	0.145	0.0357	0.0214	0.83
2862.9	11.45	16.28	5.76	7.95	50.3	48.85	0.0576	0.0295	0.00000308	0.70
2903.3	24.13	11.53	10.02	2.46	41.55	21.3	0.000001	0.000001	0.00000925	2.09
2932.8	79.23	137.33	59.91	53.99	75.62	39.31	0.00544	0.00182	0.00606	0.58
3142.8	13.69	10.85	3.9	2.25	28.46	20.76	0.00759	0.0309	0.022	1.26
3158.6	24.04	43.49	11.22	25.68	46.67	59.06	0.0156	0.00677	0.00000165	0.55
3278.2	32.81	44.23	17.11	13.32	52.14	30.11	0.0264	0.0127	0.0304	0.74
3378.8	10.44	8.52	2.42	1.91	23.17	22.39	0.0101	0.0127	0.0617	1.23
3635.2	8.72	7.44	2.04	1.32	23.38	17.81	0.022	0.0877	0.0515	1.17
3972.6	18.18	32.95	7.03	21.41	38.67	64.98	0.0227	0.000415	0.000001	0.55
4266.7	15.31	19.35	5.58	4.43	36.45	22.91	0.0193	0.0239	0.596	0.79
4282.3	13.91	34.22	6.62	35.09	47.6	102.54	0.0475	0.00283	0.000001	0.41
4400.1	8.12	9.77	2.87	2.01	35.27	20.56	0.0398	0.0182	0.0775	0.83
5064.8	9.64	13.31	4.59	4.85	47.68	36.45	0.0273	0.0117	0.00182	0.72
5094.6	4.01	3.43	1.04	0.65	25.99	19.04	0.0375	0.0862	0.0547	1.17
5263.7	4.27	3.4	1.32	0.8	30.92	23.57	0.0156	0.046	0.00304	1.26
5291.7	4.63	3.2	1.71	0.72	36.99	22.41	0.00152	0.00621	0.00142	1.45
5307.6	3.8	2.55	1.36	0.68	35.85	26.84	0.000869	0.00146	0.00177	1.49
5336.5	46.65	27.43	32.32	7.7	69.28	28.08	0.00872	0.0432	0.000001	1.70
5373.9	4.19	2.93	1.52	0.81	36.19	27.55	0.00255	0.00522	0.00177	1.43
5389.9	7.71	5.89	2.27	1.37	29.39	23.24	0.00502	0.0114	0.0737	1.31
5480.7	3.35	2.78	0.88	0.51	26.32	18.37	0.0156	0.0411	0.284	1.21
5501.1	4.96	4.05	1.35	0.94	27.19	23.34	0.0193	0.0468	0.0684	1.22
5523.4	5.51	4.65	1.52	1.08	27.58	23.17	0.0448	0.0468	0.0265	1.18
5635.9	5.01	4	1.5	0.81	30.01	20.11	0.0102	0.0239	0.000187	1.25
5721.7	6.49	5.28	1.84	1.25	28.33	23.61	0.0202	0.0368	0.0794	1.23
5753.3	11.98	8.04	6.04	2.49	50.38	30.94	0.00791	0.0319	0.00000956	1.49
5805.0	33.01	6.7	30.86	1.62	93.47	24.15	0.000492	0.000001	0.000001	4.93
5860.6	21.6	14.64	8.72	3.87	40.38	26.44	0.00233	0.0139	0.000635	1.48
5957.4	15.08	10.97	6.15	3.11	40.76	28.34	0.0089	0.0304	0.0684	1.37
5986.4	7.3	5.7	1.98	1.4	27.06	24.51	0.00759	0.0176	0.0144	1.28
6005.3	4.76	3.66	1.56	0.83	32.84	22.59	0.00759	0.0226	0.0029	1.30
6200.5	1.86	1.5	0.51	0.34	27.54	22.63	0.0133	0.0309	0.124	1.24
6227.4	8.73	6.89	2.36	1.36	26.97	19.72	0.00516	0.0139	0.013	1.27
6319.2	2.09	1.67	0.54	0.38	25.73	22.59	0.00872	0.0138	0.733	1.25
6409.8	5.38	4.23	1.38	1.02	25.69	23.99	0.00759	0.0151	0.369	1.27
6464.8	6.04	4.12	3.59	0.91	59.35	22.09	0.0175	0.0127	0.000001	1.47
6527.2	6.43	5.22	1.52	1.05	23.67	20.01	0.00759	0.0114	0.0143	1.23
6560.8	4.46	3.33	1.41	0.78	31.59	23.55	0.00392	0.00334	0.0000823	1.34
6705.7	5.49	4.27	1.84	1.14	33.48	26.63	0.0156	0.038	0.00651	1.29
6820.1	2.43	2.08	0.61	0.44	24.92	21.36	0.0392	0.154	0.191	1.17
8558.5	4.46	2.61	3.02	0.71	67.74	27.17	0.00772	0.0188	0.000001	1.71
8599.7	17.66	8.45	16.51	3.14	93.52	37.13	0.012	0.0295	0.000001	2.09

Table S2 Discriminating peaks (*P*<0.05) for BTC vs. H in discovery samples. *P* values shaded in grey were used for peak selection, where a Student t-test was used for normally distributed data (i.e. where Anderson-Darling *P* value was >0.05) and a Wilcoxon test was used for non-normally distributed data (i.e. where Anderson-Darling *P* value was <0.05). Peaks highlighted in yellow were the most significantly changing.

Mass (m/z)	Average peak area BTC +/- SD	Average peak area benign +/- SD	Fold-change (BTC vs benign)	Wilcoxon test *P* value	AUROC†
887.2	5.23 +/- 7.12	2.93 +/- 4.69	1.78	0.0243	0.74
2082.1	8.97 +/- 3.27	17.12 +/- 15.53	0.52	0.0243	0.74
2210.3	11.21 +/- 3.9	22.92 +/- 19.34	0.49	0.0243	0.75
2554.5	24.45 +/- 17.66	43.06 +/- 27.41	0.57	0.0243	0.74
2903.3	15.44 +/- 8.17	10.06 +/- 5.17	1.53	0.011	0.78
2932.8	79.15 +/- 59.89	138.59 +/- 95.83	0.57	0.0243	0.73
5805.0	39.57 +/- 35.44	21.27 +/- 20.37	1.86	0.0357	0.73

Table S3 Significant peptide peaks differentiating BTC from benign cases. †Area under the Receiver Operating Characteristic curve.

Model	Training/test set permutation	Sensitivity (%)	Specificity (%)	Accuracy (%)	PPV (%)	NPV (%)	Cross-validation (20% leave out) (%)
SVM_9pk_3knn	5	100.00	100.00	100.00	100.00	100.00	94.40%
SVM_5pk_1knn	5	100.00	100.00	100.00	100.00	100.00	93.50%
SVM_7pk_1knn	5	100.00	100.00	100.00	100.00	100.00	92.90%
SVM_9pk_1knn	5	100.00	100.00	100.00	100.00	100.00	92.90%
GA_7pk_1knn	1	100.00	100.00	100.00	100.00	100.00	92.60%
SVM_8pk_1knn	5	100.00	100.00	100.00	100.00	100.00	91.40%
SVM_6pk_1knn	5	100.00	100.00	100.00	100.00	100.00	91.10%
SVM_8pk_1knn	2	100.00	100.00	100.00	100.00	100.00	90.80%
SVM_8pk_3knn	2	100.00	100.00	100.00	100.00	100.00	90.50%
SVM_9pk_3knn	2	100.00	100.00	100.00	100.00	100.00	90.50%
SVM_auto_3knn	2	100.00	100.00	100.00	100.00	100.00	90.50%
SVM_auto_5knn	3	100.00	100.00	100.00	100.00	100.00	90.50%
SVM_3pk_1knn	5	100.00	100.00	100.00	100.00	100.00	89.90%
SVM_7pk_3knn	5	100.00	100.00	100.00	100.00	100.00	89.10%
SVM_auto_3knn	5	100.00	100.00	100.00	100.00	100.00	89.10%
GA_5pk_1knn	5	100.00	100.00	100.00	100.00	100.00	88.40%
GA_7pk_7knn	3	100.00	100.00	100.00	100.00	100.00	88.20%
GA_6pk_1knn	1	100.00	100.00	100.00	100.00	100.00	87.90%
GA_4pk_1knn	5	100.00	100.00	100.00	100.00	100.00	87.90%
GA_9pk_1knn	1	100.00	100.00	100.00	100.00	100.00	87.60%
SVM_9pk_1knn	2	100.00	100.00	100.00	100.00	100.00	87.60%
GA_4pk_1knn	2	100.00	100.00	100.00	100.00	100.00	85.80%
SVM_2pk_1knn	5	100.00	100.00	100.00	100.00	100.00	83.50%
GA_8pk_3knn	2	100.00	100.00	100.00	100.00	100.00	79.40%
SNN	5	100.00	100.00	100.00	100.00	100.00	73.80%

Table S4 Best-performing predictive models for determining BTC versus healthy. A SVM 9 peak model was the best performing on cross-validation and for test set classification (shaded grey).

Ave Mass by MALDI-TOF (m/z)	Calculated average mass (Da)	Calculated average mass $[M+H]^+$	Calculated monoisotopic mass (Da)	Name	Sequence	Identification
887.2						no
1020.2	1020.11	1021.12	1019.50	Fibrinopeptide A	DFLAEGGGVR	yes; Villanueva et al; Tiss et al
1263.7	1263.33	1264.34	1262.59	Fibrinopeptide A	GEGDFLAEGGGVR	yes; Villanueva et al; Tiss et al
1350.8	1350.41	1351.42	1349.62	Fibrinopeptide A	SGEGDFLAEGGGVR	yes; Villanueva et al; Tiss et al
1363.4						no
2082.1	2086.07	2087.08	2084.89	Fibrinogen alpha	GGSTSYGTGSETESPRNPSSAG	Koomen et al
2210.3	2209.33	2210.34	2208.05	HMW kininogen	KHNLGHGHKHERDQGHGHQ	Villanueva et al; Tiss et al
2554.5	2553.59	2554.60	2552.09	Fibrinogen alpha	SSSYSKQFTSSTSYNRGDSTFES	Villanueva et al; Tiss et al
2624.6	2624.93	2625.94	2623.40	Fibrinogen alpha	LVTSKGDKELRTGKEKVTSGSTTTT	Koomen et al
2903.3						no
2932.8	2932.02	2933.03	2930.28	Fibrinogen alpha	SSSYSKQFTSSTSYNRGDSTFESKSY	Villanueva et al; Tiss et al
5064.8						no
5805.0	5805.09	5806.10	5801.63	Fibrinogen alpha	SSSYSKQFTSSTSYNRGDSTFESKSYK MADEAGSEADHEGTHSTKRGHAKSRP	yes

Table S5 List of identified and predicted peak identifications. C18-extracted and gel separated serum samples were subjected to high-mass accuracy LC-MS/MS as described in Materials and Methods. Predicted identifications were made from published lists of MALDI-TOF and LC-MS/MS peak identifications, including our own previous work (Ref 17; Tiss et al.).

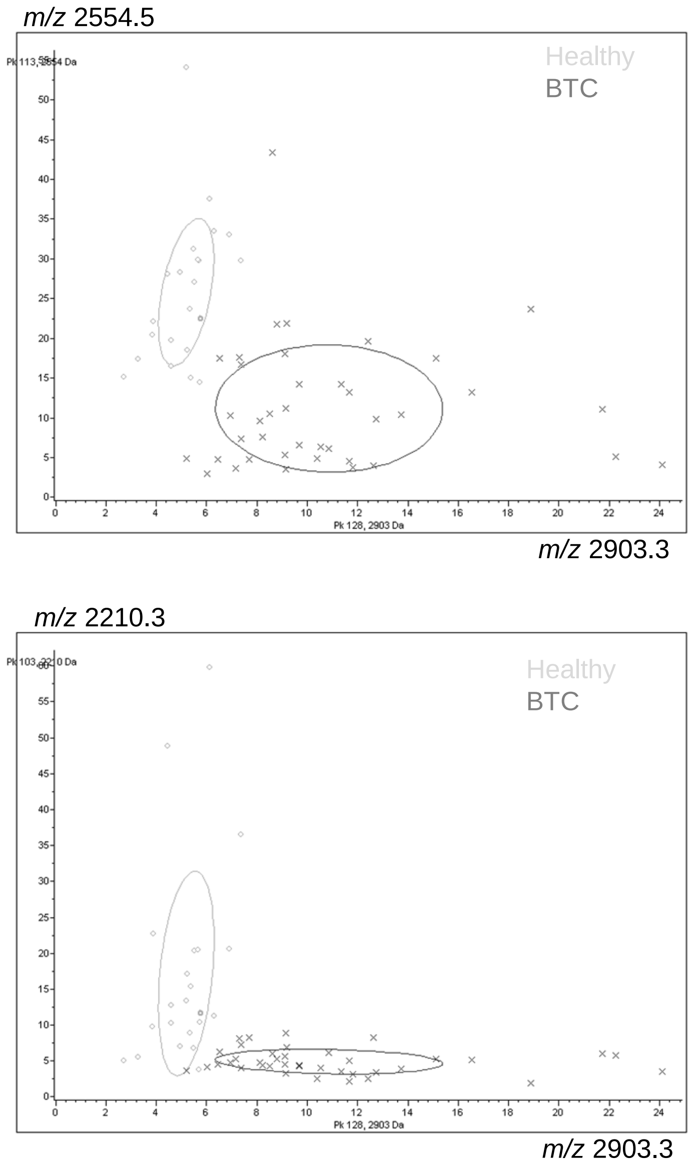

Figure S1 2D scatter plots of peak intensities for **A.** *m/z* 2554.5 vs *m/z* 2903.3 and **B.** *m/z* 2210.3 vs *m/z* 2903.3 showing separation of BTC and healthy samples.

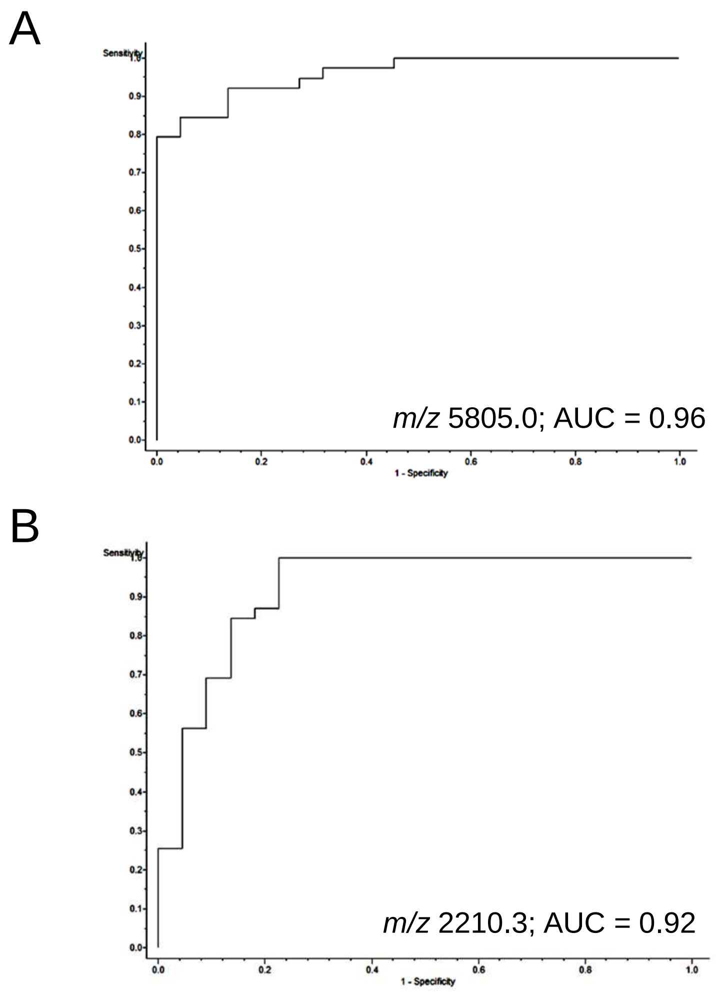

Figure S2 Receiver Operating Characteristics (ROC) curve analysis. **A.** The peak at *m/z* 5805.0 gave an area under the curve (AUC) of 0.96 for BTC versus healthy samples. **B.** The peak at *m/z* 2210.3 gave an AUC of 0.92 for BTC versus healthy samples.

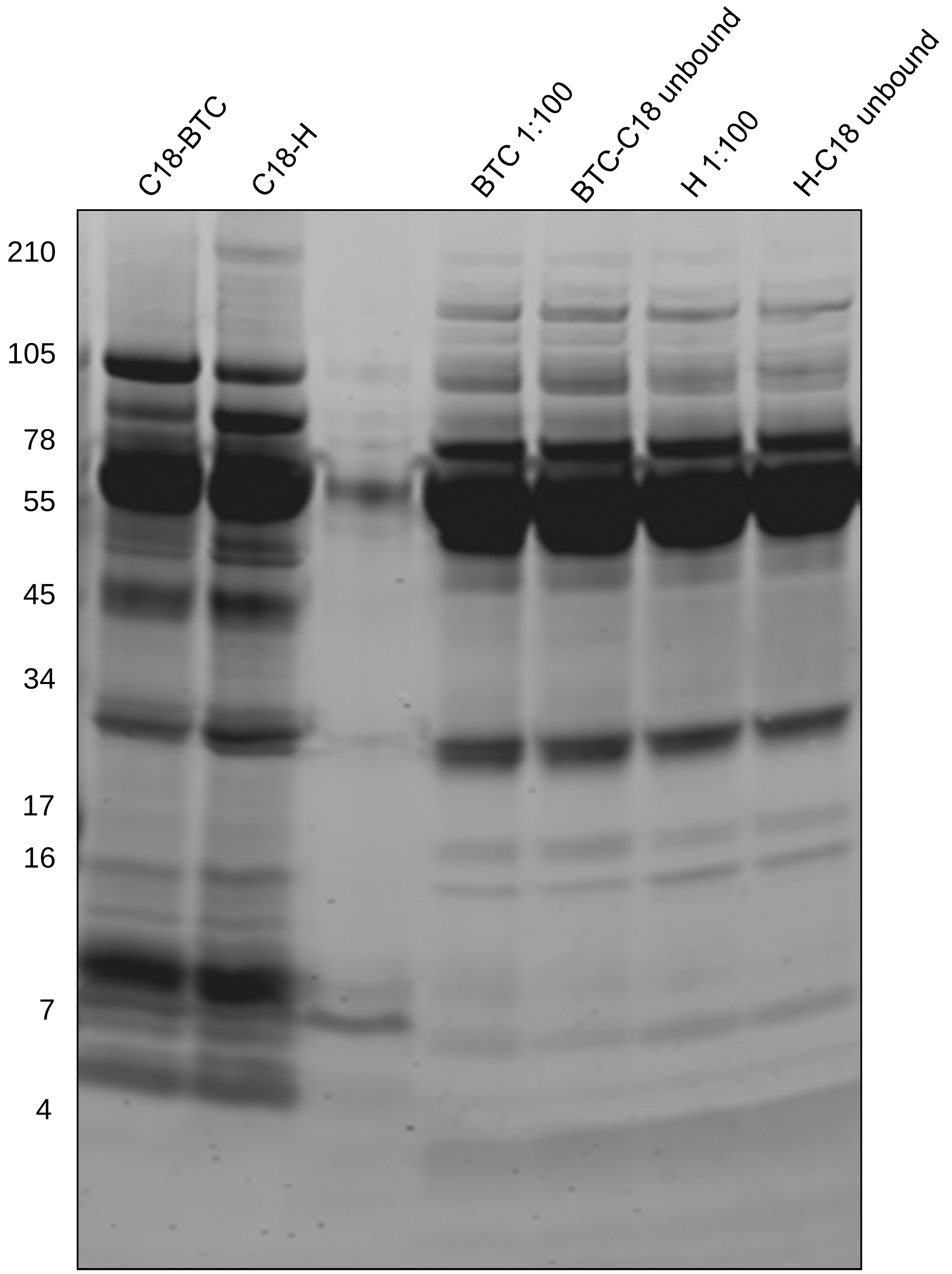

Figure S3 1D-Tris-tricine (10-20%) gel of C18 bead-extracted sera, unbound material and crude sera. Protein loads (~15 µg) were C18-extracted BTC sample pool (C18-BTC), C18-extracted healthy sample pool (C18-H), crude BTC serum (BTC 1:100), crude healthy serum (H 1:100;), unbound BTC material (BTC-C18 unbound) and unbound healthy material (H-C18 unbound). Bands below 20 kDa were cut from the gel, proteins/peptides passively eluted and analysed by LC-MS/MS.
